# H7N9 and H5N1 avian influenza suitability models for China: accounting for new poultry and live-poultry markets distribution data

**DOI:** 10.1007/s00477-016-1362-z

**Published:** 2016-12-05

**Authors:** Jean Artois, Shengjie Lai, Luzhao Feng, Hui Jiang, Hang Zhou, Xiangping Li, Madhur S. Dhingra, Catherine Linard, Gaëlle Nicolas, Xiangming Xiao, Timothy P. Robinson, Hongjie Yu, Marius Gilbert

**Affiliations:** 1 Spatial Epidemiology Lab. (SpELL), Université Libre de Bruxelles, Brussels, Belgium; 2 Division of Infectious Disease, Key Laboratory of Surveillance and Early-warning on Infectious Disease, Chinese Center for Disease Control and Prevention, 155 Changbai Road, Changping District, Beijing, 102206 China; 3 WorldPop, Department of Geography and Environment, University of Southampton, Southampton, SO17 1BJ UK; 4 Key Laboratory of Public Health Safety, Ministry of Education, School of Public Health, Fudan University, Shanghai, 200032 China; 5 Institute of Biodiversity Science, Fudan University, Shanghai, 200433 China; 6 Department of Animal Husbandry & Dairying, Government of Haryana, Pashudhan Bhawan, Bays No. 9-12, Sector -2, Panchkula, Haryana 134109 India; 7 Department of Geography, Université de Namur, Namur, Belgium; 8 Department of Microbiology and Plant Biology, Center for Spatial AnalysisUniversity of Oklahoma, 101 David L. Boren Blvd, Norman, OK 73019 USA; 9 Livestock Systems and Environment (LSE), International Livestock Research Institute (ILRI), Nairobi, Kenya; 10 Fonds National de la Recherche Scientifique, Brussels, Belgium

**Keywords:** Poultry data, Avian influenza, Spatial epidemiology, HPAI H5N1, LPAI H7N9

## Abstract

In the last two decades, two important avian influenza viruses infecting humans emerged in China, the highly pathogenic avian influenza (HPAI) H5N1 virus in the late nineties, and the low pathogenic avian influenza (LPAI) H7N9 virus in 2013. China is home to the largest population of chickens (4.83 billion) and ducks (0.694 billion), representing, respectively 23.1 and 58.6% of the 2013 world stock, with a significant part of poultry sold through live-poultry markets potentially contributing to the spread of avian influenza viruses. Previous models have looked at factors associated with HPAI H5N1 in poultry and LPAI H7N9 in markets. However, these have not been studied and compared with a consistent set of predictor variables. Significant progress was recently made in the collection of poultry census and live-poultry market data, which are key potential factors in the distribution of both diseases. Here we compiled and reprocessed a new set of poultry census data and used these to analyse HPAI H5N1 and LPAI H7N9 distributions with boosted regression trees models. We found a limited impact of the improved poultry layers compared to models based on previous poultry census data, and a positive and previously unreported association between HPAI H5N1 outbreaks and the density of live-poultry markets. In addition, the models fitted for the HPAI H5N1 and LPAI H7N9 viruses predict a high risk of disease presence for the area around Shanghai and Hong Kong. The main difference in prediction between the two viruses concerned the suitability of HPAI H5N1 in north-China around the Yellow sea (outlined with Tianjin, Beijing, and Shenyang city) where LPAI H7N9 has not spread intensely.

**Electronic supplementary material:**

The online version of this article (doi:10.1007/s00477-016-1362-z) contains supplementary material, which is available to authorized users.

## Introduction

In high-income countries, most of the intensification of poultry production took place in the second half of the 20^th^ century and are not changing much anymore (FAO 2009). Hotspots of intensive poultry production can be found in several states of the USA and in north-western Europe (Robinson et al. 2014), where they have been present for decades and will probably remain so for a number of years. In contrast, transition economies such as China or Brazil are intensifying their animal production in response to rising demands from more urbanized and wealthy human populations that are increasing their per capita consumption of poultry meat and eggs (Robinson and Pozzi 2011). Therefore, both the number and geographical distribution of poultry is changing much faster than that in high-income economies. This has two main consequences. First, changes in densities and geographical distribution influence the conditions of spread and evolution of infectious diseases. Higher densities generally translate into higher contact rates between animals, which, alongside other mechanisms, may explain why several disease emergences were linked to recent intensification of livestock production systems (Jones et al. 2013). Secondly, the density of host is a key variable in any epidemiological investigations, and studies carried out in economies with a fast-changing agricultural sector need to account for those changes.

China is specifically in this situation. In the last 20 years, the stock of chickens and ducks was multiplied by a factor of 2.61 and 2.36, respectively, corresponding to a compounded annual growth rate of 3.91 and 2.36% (FAO 2009). The country now holds by far the largest population of chickens, and 70% of the world’s ducks. It is in this context of intensification of poultry production and fast environmental and land-use changes (Wei and Ye 2014) that two major avian influenza viruses (AIVs) infecting humans emerged in the country. In 1996, the highly pathogenic avian influenza virus (HPAIV) H5N1 was first reported in southern China (Li et al. 2004) and for several years was only found in that country. In 2003–2004, it started to spread to other countries and reached a maximum geographical range in 2006, when a cumulative number of over 60 countries had reported the presence of the virus across Asia, Europe and Africa (Hogerwerf et al. 2010). In 2013, new infections caused by a low pathogenic avian influenza virus (LPAIV) H7N9 were notified in humans, and the infections were traced back to live-poultry markets (Cowling et al. 2013; Yu et al. 2013). The H5N1 and H7N9 viruses had very different pathogenicities in poultry and both showed the capacity to infect humans, though with somewhat different epidemiological characteristics (Cowling et al. 2013; Qin et al. 2015).

Surprisingly, although chicken and duck are so critical as potential driver of disease emergence or as separate epidemiological variables in risk-factor analyses, separate statistics on their distribution are not routinely produced with a high spatial level of details in China and there exists a great degree of heterogeneity in reporting and aggregation of census data (Prosser et al. 2011). Data are collected at a fine level (typically counties) through censuses or surveys, but when these data become centralized at the prefecture (administrative level 2) or province level (administrative level 3), details are lost, either because of spatial aggregation (e.g. provincial yearbook only reporting prefecture-level aggregated data, or national yearbook only reporting province-level data), or because of thematic aggregation with chicken and ducks being pooled together in a “poultry” category. So, many country-level data are currently not centralized in a high-resolution spatial database, although they may be collected on the ground and both the spatial and temporal resolution of database can be a limiting factor for epidemiological investigation in a fast-changing sector.

Previous investigations strongly demonstrated the need to separate domestic waterfowls (ducks and geese) from gallinaceous poultry as their association with HPAI H5N1 virus presence was consistently found to differ in several Asian countries (Gilbert et al. 2006, 2008; Gilbert and Pfeiffer 2012). In addition, different types of production systems, such as extensive backyard production on the one hand and more commercial modes of production on the other, may also have different types of influence on transmission risk. Recent work on HPAI H5N1 and on LPAI H7N9 found different patterns of association between extensively and intensively raised ducks and chickens in Thailand (Van Boeckel et al. 2012) and China (Gilbert et al. 2014). So, there is a strong need for analysing both HPAI H5N1 and LPAI H7N9 data in relation to detailed and up to date separate, duck and chicken data, in addition to making a distinction between extensive and intensive production systems.

Several studies have previously investigated the spatial distributions in China of both HPAI H5N1 (Li et al. 2015b; Martin et al. 2011a; Fang et al. 2008) and LPAI H7N9 (Gilbert et al. 2014; Fang et al. 2013). In a recent study, the spatial distribution of human infections with HPAI H5N1 and H7N9 was studied and compared, which yielded insights on the areas of co-circulation and potential infections (Li et al. 2015c), and in another study with a strong emphasis on climatic factors (Li et al. 2015a). However, due to the limited poultry data availability highlighted above, few of these studies made a distinction between extensive and intensive systems, and several included a general “poultry” category that does not differentiates chicken from ducks.

In this paper, we aimed to revisit and update previous HPAI H5N1 and LPAI H7N9 suitability models using a novel and improved set of poultry data in China separating chicken and ducks and different production systems with state-of-the-art recent downscaling methodology (Nicolas et al. 2016). In addition, we also tested live poultry market density as predictor of HPAI H5N1 outbreaks distribution, which was not done in previous studies due to the lack of available census data at the time of previous studies. So, the specific objectives were to compare the result obtained with the updated poultry data sets, to test the effect of live-poultry market density on HPAI H5N1 distribution, and to compare the geographical distribution of high HPAI H5N1 and LPAI H7N9 suitability within China.

## Materials and methods

### Poultry statistics

Census data on chicken and duck numbers at the end of the calendar year 2011 and 2012, and the numbers of individuals sold per year were obtained from three sources: (a) published yearbooks, such as the China Animal Husbandry Yearbook, Statistic Yearbook of China or provincial yearbooks (e.g. http://data.stats.gov.cn/); (b) the official website of the Ministry of Agriculture of China (http://english.agri.gov.cn/) and the Agricultural Bureaus at province and prefecture level; (c) contact with provincial Bureaus of Animal Husbandry, provincial Departments of Commerce, Statistics Bureaus and Chinese Agricultural Universities to obtain any data not available from sources (a) or (b). These data were mostly available at the prefecture level (administrative level 2) but not consistently for all prefectures and provinces. For example, a prefecture could have a certain value in the 2011 data set, but not in the 2012 one. For a few provinces, namely Anhui, Jiangsu and Zhejiang in Eastern China, we were also able to find county-level data for the year 2010.

Despite the exploration of the available above data sources, we were not able to find 2010, 2011 or 2012 data for all provinces and prefectures. In a previous study, Prosser et al. (2011) already created a composite poultry dataset for chicken, duck and geese, based on census statistics from 2003 and 2005, and this composite data set remains to date the basis of the poultry data for China in the Gridded Livestock of the World (GLW) database. The term ‘composite’ refers to the fact that they already had to combine data from different spatial scale: province, prefecture and counties. Therefore, we combined our recent data set with the one from Prosser et al. (2011) in case of complete absence of information in the concerned area. Namely, for each of the counties in China, we checked different available data sources and used the density data from the source with the following order of priority: (i) a 2010 value from the county-level data set (available only in Anhui, Jiangsu and Zhejiang), (ii) a 2011 or 2012 value from the prefecture-level data set, and (iii) a value from the Prosser et al. (Prosser et al. 2011) data set. In addition, when an administrative level 2 (prefecture) count was available in 2011 or 2012 in an area that had administrative level 3 (counties) data in the Prosser et al. (Prosser et al. 2011) data set, we used the county-level data corrected to match the 2011 or 2012 prefecture counts by multiplying them by a single scalar. In order to account for differences in reference years, we applied national-level growth correction factors from FAOSTAT (FAO 2009) to account for the differences in years, with 2010 as pivot year. A similar approach was used for both chicken and ducks. Due to a lack of data for geese, we did not consider that species in further processing.

### Poultry downscaling

In order to avail the poultry data at the same spatial resolution as other risk factors, we used the downscaling methodology of the Gridded Livestock of the World (GLW), which was fully described in Robinson et al. (Robinson et al. 2014) and Nicolas et al. (Nicolas et al. 2016). This method relies on models being built based on the census counts and a set of covariates, and typically models species at the continental level (Robinson et al. 2014; Nicolas et al. 2016). Therefore, models can be trained using the data outside a specific country. In order to benefit from training chicken and duck data in countries near China, we extracted polygons with chicken and duck census counts from the GLW within an arbitrarily 500 km buffer from China’s border, to benefit from good quality input data from areas with similar agro-ecological conditions. The GLW methodology is only briefly summarized here. First, the density of animals per km^2^ of suitable land is estimated in all polygons corresponding to the sub-national poultry census data and transformed to its logarithmic value (base 10). Second, a large set of sample points is built to cover the modelling extent, and values for the observed densities and predictor variables were extracted from their respective polygons (census data) or pixels (predictor variables). Third, the sample file was divided into *n* sub-samples for bootstrapping the analysis, and each sub-sample file was divided in two parts, one for building the model with 70% of the polygons, and one for evaluating the model goodness of fit with 30% of the polygons. Fourth, each sub-sample was used to build a Random Forest model and the model was applied to the raster imagery to obtain a single predicted value for each pixel. Fifth, the predicted values were averaged over the *n* bootstraps. Finally, post-processing was carried out to correct pixels values by multiplying all pixels of a particular census polygon by a constant so that the sum of the grid cell values within the polygon was equal to the observed totals in the input subnational census data. Finally, the pixel values were also corrected so that the national total matches the FAOSTAT official total for a specified base year, in this case 2010. The spatial covariates used to make the predictions include Fourier-transformed remotely sensed variables (the normalized difference vegetation index and enhanced vegetation index, the day and night land surface temperature and the band 3 shortwave infrared band), eco-climatic variables (length of growing period and annual precipitation), topographic variables (elevation and slope) and anthropogenic variables (human population density and travel time to major cities). We used exactly the same set of predictor variables as described in Robinson et al. (Robinson et al. 2014) and Nicolas et al. (Nicolas et al. 2016), with the exception of the human population density, where the recent 2010 human population data published for China by the Worldpop project was used (Gaughan et al. 2016). The chicken density layer was finally broken down between extensively and intensively raised chickens, following the methodology outlined in Gilbert et al. (Gilbert et al. 2015).

### Avian influenza data

For the HPAI H5N1 models, we used the outbreak locations from the epidemiological dataset described in Martin et al. (Martin et al. 2011a), complemented by outbreak locations extracted from the FAO Empres-i data base, including 76 recent records (from 2009 to 2016) of HPAI H5N1 infection in domestic poultry (Claes et al. 2014). Boosted regression trees (BRT) models require data on both presence and absence and pseudo-absences were generated throughout the country in locations: (1) where there was no evidence of previous HPAI H5N1 presence; (2) at a minimum distance of 0.0833 decimal degree of any positive (which correspond to the spatial resolution of the poultry density layer); and (3) in a location where human and poultry density was higher than five person or bird per km^2^ to exclude desert, unpopulated areas and areas with potentially very low surveillance from the analysis. There is no consensus on the optimal number of pseudo-absences to be used in niche modelling methods (Barbet-Massin et al. 2012). This number depends of the species under consideration, type of model used and the spatial extent of data. Pseudo-absences were generated in much greater numbers than HPAI outbreaks (eight times more negatives than positives) to capture enough variability in data and optimizing the model performance.

For the LPAI H7N9 models, we used the set of positive and negative markets described in Gilbert et al. (Gilbert et al. 2014), complemented by H7N9 presence location recorded by China CDC up to 01/10/2015 (first three epidemic waves). As the epidemiological unit is the market and the data set included absence points (markets where H7N9 was never recorded), there was no need to distribute pseudo-absences. The spatial locations of markets described above was used to create a layer of market density (market/km^2^) on a grid of 0.0833 decimal degree of resolution.

### Avian influenza suitability modelling

Boosted regression tree (BRT) models were used to model the probability of presence of HPAI H5N1 outbreaks at the pixel level and probability of LPAI H7N9 infection at markets level (Elith et al. 2008). The method is increasingly used in suitability modelling of infectious (Gilbert et al. 2014; Pigott et al. 2014) and vector-borne diseases (Bhatt et al. 2013) for its capacity to model interactions between variables as well as non-linear relationships between the outcome and predictor variables. Each model was evaluated with an eightfolds cross-validation procedure and the area under the receiver operating characteristic curve used as a measurement of the discriminatory capacity of models (Elith et al. 2008). The data set is split in 8 sub-data and a single sub-data is retained as the validation data and the remaining 7 sub-data are used as training data. The cross-validation is then repeated 8 times for each BRT model. Finally, in order to account for sources of uncertainty in the model (on the localisation of pseudo-absences and the data splitting of cross-validation), the analysis was bootstrapped with 15 independent BRT run for a total of 120 cross-validations (15 runs × eightfolds).

In order to ensure comparability with the previously published results, we used a similar set of predictor variables as in Martin et al. (Martin et al. 2011a) and Gilbert et al. (Gilbert et al. 2014), including the chicken and duck density data layers produced by the processing detailed in the previous section, the market density layer (Gilbert et al. 2014), human population density from the Worldpop project (www.worldpop.org) (Gaughan et al. 2016), the proportion of land covered by water and the proportion of land covered by rice cropping from the GlobCover database (Bicheron et al. 2008) and the cropping intensity established through remote sensing (Xiao et al. 2005, 2006). So, the final set of predictor variables included: extensively raised chicken density (ChExtDn, heads/km^2^), intensively raised chicken density (ChIntDn, heads/km^2^), duck density (heads/km^2^), live-poultry market density (MktDn, markets/km2), human population density (HpopDn, people/km^2^), the cropping intensity (CropInt, crop cycles/year), the proportion of area covered by rice paddy fields (RiceCov, %) and the proportion of area covered by permanent water (WatCov, %). Since a market’s epidemiological situation reflects the potential circulation of viruses in its catchment areas, a spatial filter was applied to each predictor variable, following the procedure outlined in Gilbert et al. (Gilbert et al. 2014). In this step, the covariate values were smoothed with weights determined by a Gaussian kernel and the parameter σ representing, the size of the catchment area. A range of values for the parameter σ was tested in Gilbert et al. (Gilbert et al. 2014) and the same figure was used in this study (σ = 0.7). In addition, Gilbert et al. (2014) used modelled live-poultry market densities as predictor variables in their model, so that they could extrapolate their model to the rest of Asia. For the sake of comparability, we used the same layer, but the raw observed number of live-poultry market by pixel was also tested to evaluate the potential effect of the modelling procedure.

## Results

**Fig. 1:**
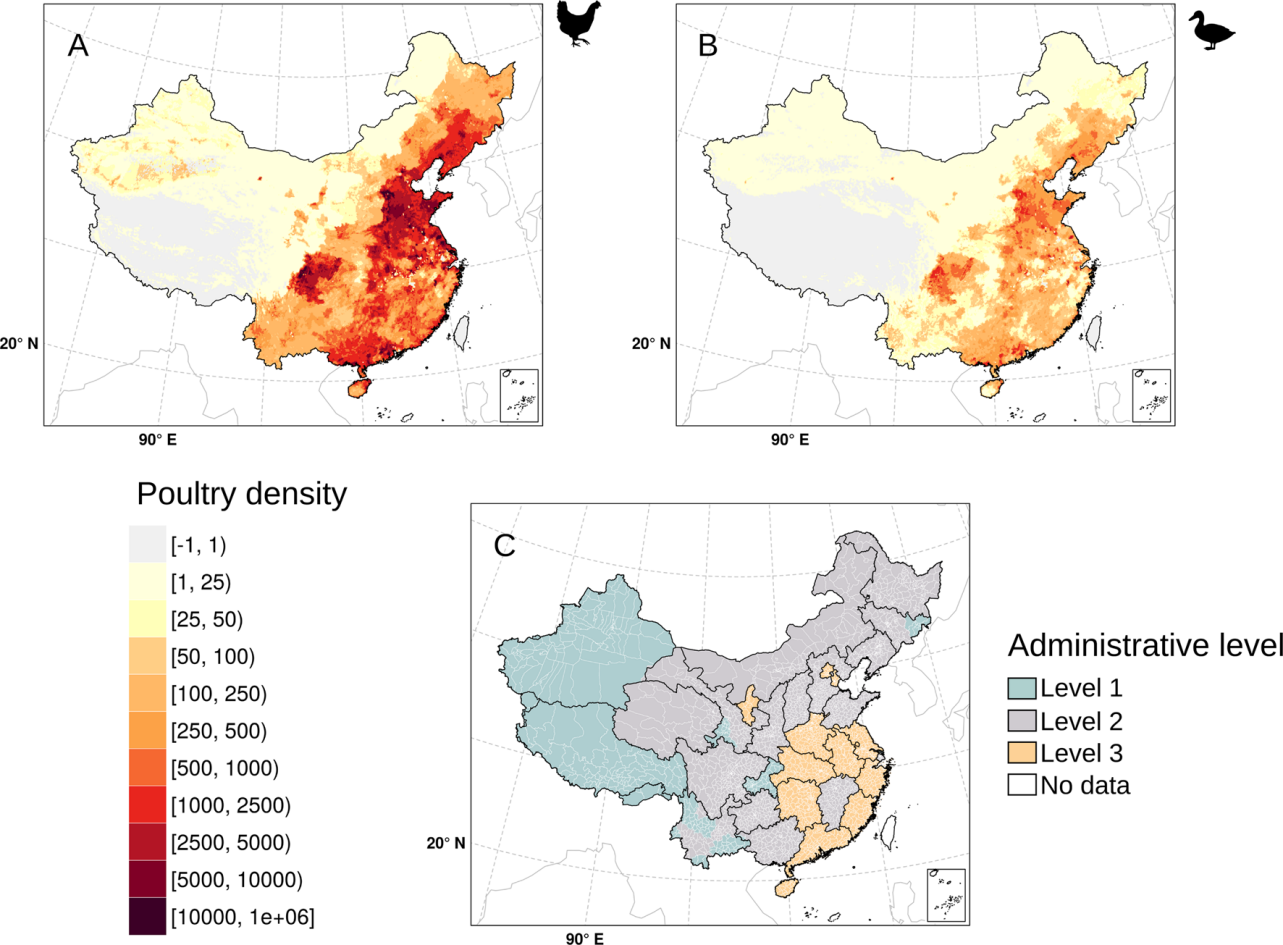
Overview of base poultry data resolution. The new data sets of chicken (**a**) and duck (**b**) density (heads/km^2^—on a logarithmic scale of base 10) are obtained by combining recent census data sets at different spatial levels (**c**; level 1: province; level 2: prefecture; level 3: county). This figure was built with the R-3.3.1 software (https://cran.r-project.org/). The graticule is composed of a 10-degree increments and the coordinate system is ‘SR-ORG:7564’

**Fig. 2:**
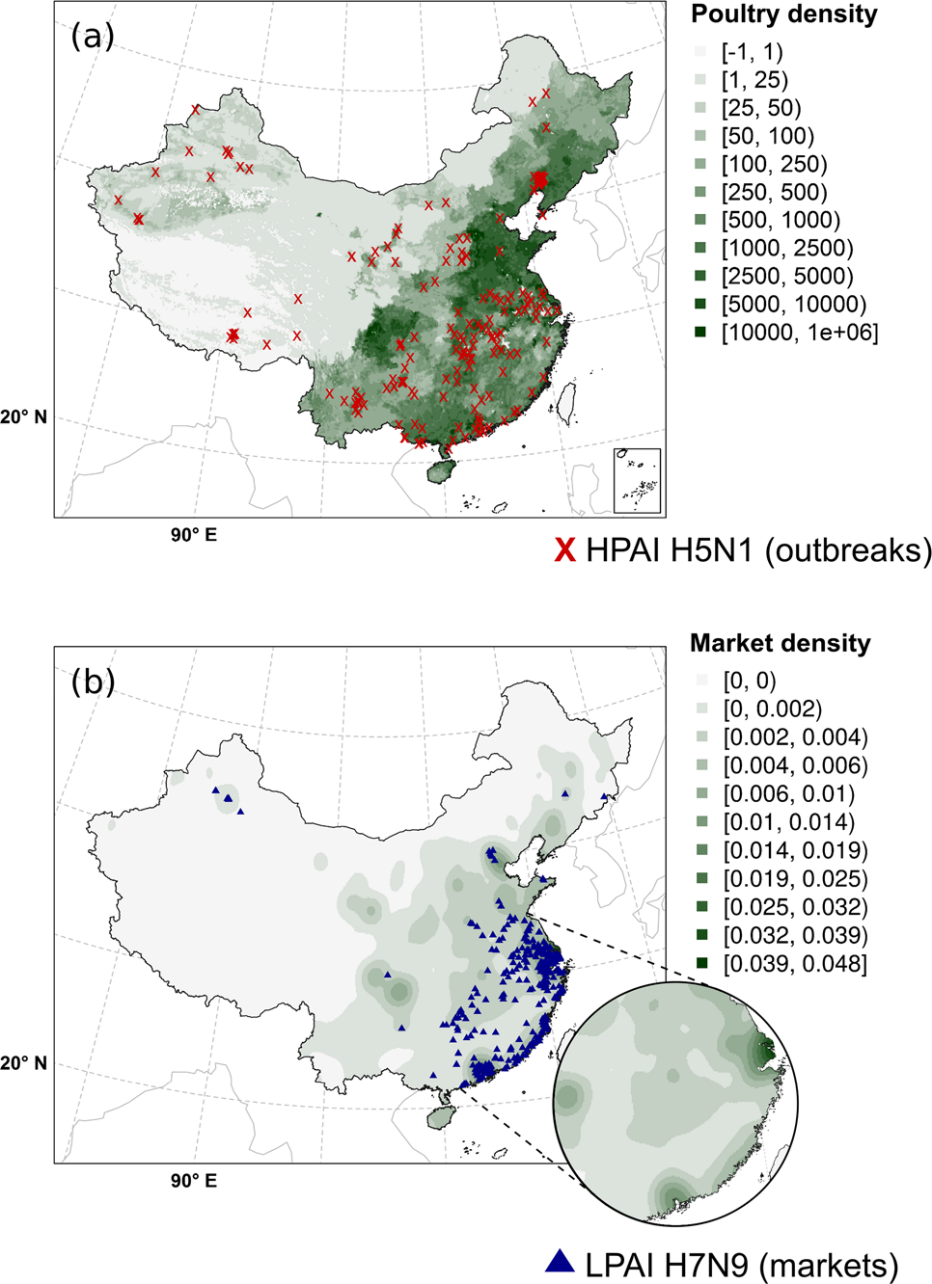
Distribution of HPAI H5N1 outbreaks (**a**; *red cross*) and LPAI H7N9 infected markets (**b**; *blue triangles*) in China included in this study. The density of poultry (the sum between the chicken and the duck density) and live bird markets (smoothed) are also displayed in the maps (**a**) and (**b**) respectively. The density of live bird markets was smoothed with weights determined by a Gaussian kernel and the parameter σ representing the size of the catchment area (σ = 0.7). This figure was built with the R-3.3.1 software (https://cran.r-project.org/). The graticule is composed of a 10-degree increments and the coordinate system is ‘SR-ORG:7564’

The new poultry distribution is displayed in Fig. 1, showing generally much higher densities of chicken over ducks, along with the final level at which input census data was available. The distribution of ducks in China largely follows that of chickens, with some of the highest densities observed in the northeastern provinces of Shanxi, Heibei, Shangdong, south of Beijing, and the southern provinces of Guangxi and Guangdong. In central China, the Sichuan province stands out as having particularly high densities too. Figure 2 displays the density of poultry and live bird markets with the distribution of HPAI H5N1 and LPAI H7N9 cases. The live bird market density is high on the east coast of China and inland, around some cities as Chongqing and Lanzhou.

**Fig. 3:**
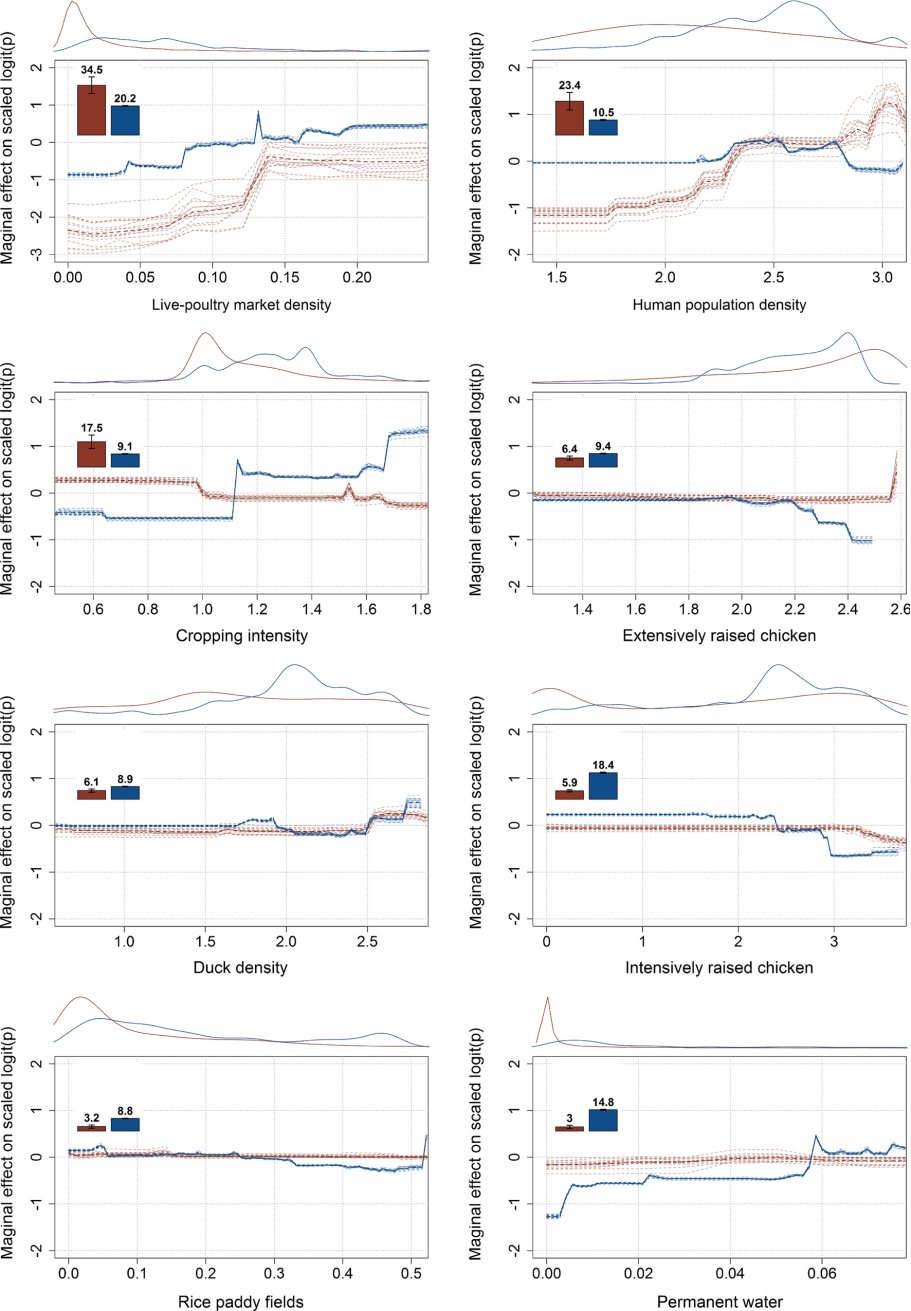
Relative contribution (*bar plots*) and partial dependent plot (*curves*) of each predictor of the BRT models of HPAI H5N1 outbreaks (*red*) and LPAI H7N9 infected markets (*blue*). The relative contribution of each predictor is scaled so that the sum of all predictor variables adds to 100%, and measures the number of times a predictor is selected for splitting the dataset over the trees. The partial dependent plot gives a graphical description of the marginal effect of a predictor on the predicted response. The opaque line represents the mean marginal effect, whilst transparent lines represent each bootstrap. On the *top* of each graph, the density function of the observed distribution of predictors is displayed for one bootstrap and for the two analyses (*red* HPAI H5N1; *blue* LPAI H7N9)

The relative influences in the BRT models of the predictor variables as well as their profiles are displayed in Fig. 3. For HPAI H5N1 outbreaks, the variables with the highest relative contribution (RC) were the live-poultry market density (positive association, RC 34.5%), human population density (positive association, RC 23.4%), and cropping intensity (negative association, RC 17.5%). One should note that when the raw number of live-poultry markets is used as predictor instead of the one modeled in Gilbert et al. (2014), both the human population density and live-poultry market density remain the most important factors, but their respective relative contribution is inverted, i.e. human population density has the highest RC (SI 1 Fig A, B).

For LPAI H7N9 infected markets, the predictor variables with the highest relative contributions were the poultry market density (20.2%) followed by the density of intensively raised chicken (18.4%), the proportion of land covered by water (14.8%) and human population density (10.5%). Interestingly, when repeating the analysis by breaking down LPAI H7N9 records by seasonal epidemic waves, the relative contribution of live-poultry market density in the LPAI H7N9 market models tended to decrease over time, with values of 22.5, 17.7 and 11.7 for the 2012/2013, 2013/2014 and 2014/2015 winters epidemic waves, respectively (SI 2 Fig. A). Markets reporting LPAI H7N9 virus infections are associated positively with the proportion of area covered by water and the market density as observed in the BRT profiles of Fig. 3. The opposite trend is observed for the chicken density layers, which are negatively associated with LPAI H7N9 presence. The effect of using the raw number of live-poultry market per pixel instead of the modeled one was a reduction in its RC from 20.2 to 17.9, and it remained the top predictor in terms of RC.

**Fig. 4:**
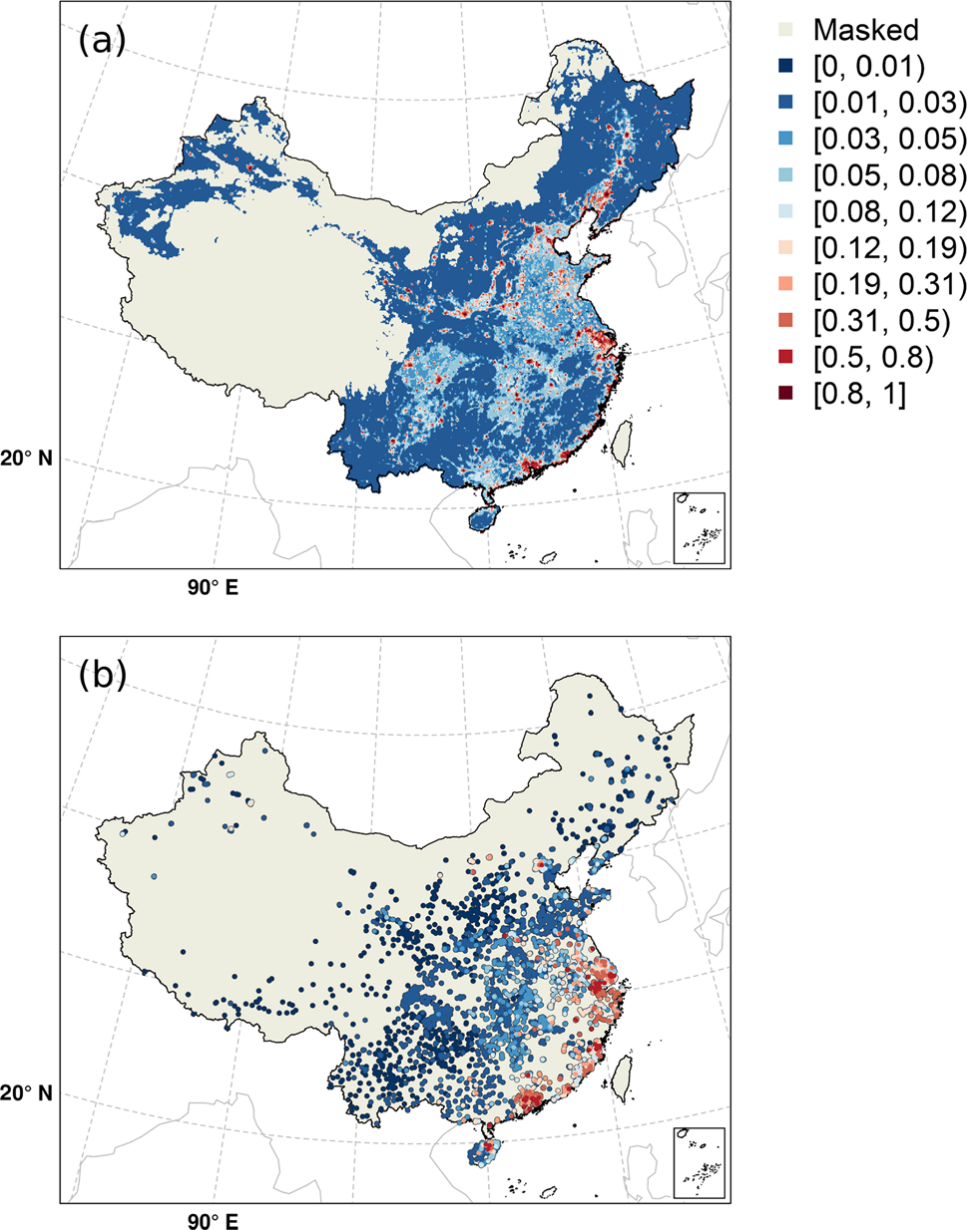
Predicted maps of the probability of presence of HPAI H5N1 outbreaks (*top*) and the probability for a market of being infected by LPAI H7N9 (*bottom*). Note that infection risk is estimated as the probability that a pixel (HPAI H5N1) or market (LPAI H7N9) would be infected. The mask corresponds to the areas where human and poultry density was lower than five persons, heads/km^2^. This figure was built with the R-3.3.1 software (https://cran.r-project.org/). The graticule is composed of a 10-degree increments and the coordinate system is ‘SR-ORG:7564’

HPAI H5N1 and LPAI H7N9 suitability maps are displayed in Fig. 4a and b, respectively. While LPAI H7N9 remained constrained to the two hotspots of Shanghai and Guangdong, coastal areas and a number of small and isolated pockets with higher suitability in and around inland cities, HPAI H5N1 probability of presence was found to be distributed over more widespread zones in inland China, with much higher probabilities of presence in large rural areas. Both models had comparable goodness of fit measurements, with AUC values of 0.885 ± 0.039 and 0.850 ± 0.024 for the HPAI H5N1 and LPAI H7N9 models respectively.

## Discussion

Overall, the changes due to the effect of the improved poultry variables were relatively limited, and provided several results consistent with the previous analyses (Martin et al. 2011a; Gilbert et al. 2014). For HPAI H5N1 outbreaks, this involved a predominance of anthropogenic factors (human population density or live poultry market density) with a relatively limited influence of poultry variables. One can note, however, a slight increase in the marginal effect linked to the highest duck densities (>300 ducks/km^2^; ~2.48 on a logarithmic scale) and a decrease linked to the highest densities of intensively raised chickens (>1500 chickens/km^2^; ~3.18 on a logarithmic scale). For LPAI H7N9, the results are also fairly similar, and focusing on changes in the effect of the poultry factors, we note a slightly higher RC of duck density, with a higher marginal effect linked to duck densities >300 ducks/km^2^, and the confirmation of the negative association with intensively and extensively raised chicken. It is in fact quite surprising not to find a stronger association between HPAI H5N1 outbreaks and different poultry variable, as such association have consistently been found elsewhere. It is hard to say if this relates to a yet unsatisfying quality of the poultry data, differences in reporting, or whether this may be due to a true absence of statistical association with data adequately reflecting the situation on the ground.

One noticeable change to the HPAI H5N1 model was the inclusion of the live-poultry market density, which was found to be a strong predictor of HPAI H5N1 presence, when used in its modelled (RC = 34.5%) or raw (RC = 17.9%) form. The distribution of live-poultry markets and human population are strongly correlated, so their respective effects are in fact quite difficult to separate on a purely statistical ground. However, the fact that both appeared as strongly significant suggests that they may exert simultaneous influences. This statistical association fits with many recent results on live-poultry market networks highlighting their possible key role in HPAI H5N1 persistence. This was suggested by simulation studies (Fournié et al. 2013) and social-network analysis of live-poultry market networks in China (Martin et al. 2011b) and Vietnam (Magalhães et al. 2010; Fournié et al. 2012) but was never quantitatively demonstrated on such a large spatial scale. One should note that this was made possible thanks to the important effort of collecting live-poultry market census data following the emergence of LPAI H7N9 (Gilbert et al. 2014). Many countries where HPAI H5N1 persisted over long periods of time have a large part of their poultry being traded through live-poultry markets, including China, Vietnam, Indonesia, Bangladesh or Egypt. By contrast, Thailand, for example, which presents all risk factors usually associated with high HPAI H5N1 risk such as high free-grazing duck density, dense irrigated areas, co-existing extensive and intensive poultry systems and human population density, has very few live-poultry markets, for cultural reasons, and this may contribute to explain the success of the country in eradicating the disease. Our results support the suggestions made recently by several authors that focusing surveillance and control in markets and adapting their management to include cleaning and/or closing day might be the key to preventing HPAI H5N1 persistence too (Fournié et al. 2011, 2013).

Interestingly, we also tentatively showed that the relative contribution of live-poultry market density to the LPAI H7N9 market models reduced over time, and this pattern is observed alongside an overall reduction in the number of human cases noted in the last years. The specific objective of this analysis was not to make a full assessment of the model over time, which could be explored in future works. However, they may support the hypothesis that enhanced surveillance, control and management in markets after the first epidemic wave may have reduced their role in disease transmission. For example, previous papers have already showed a clear association between the timing of market closure and reductions in human cases (Yu et al. 2013; Wu et al. 2014). Our live-poultry market database does not take markets closures or opening status into account, and some markets may have changed their management practices, disappeared or have been closed over time. For example, much trading of live-poultry was banned in markets in the periphery of Beijing, but this was not accounted for in our model, which still highlights Beijing as being a potential local hotspot due to its high density of markets in our dataset.

In terms of geographical distributions, the resulting HPAI H5N1 suitability map reflects the higher contribution of live-poultry market density by producing a much more clustered distribution of suitability than that predicted by Martin et al. (2011a). Our HPAI H5N1 outbreak suitability map does not overlap so much with the recent H5N1 human infection risk map produced by Li et al. (2015c), who highlighted high risk regions for human infections as being mostly concentrated in southern China. This may be partly explained by the difference in outcome: they studied the distribution of human cases and we investigated the distribution of outbreaks in poultry. However, this does not entirely explain why there would be fewer human cases in Northern China if the landscape is suitable for H5N1 infections in poultry and if outbreaks were reported there. However recent work comparing the epidemiology of H7N9 and H5N1 viruses suggests that the susceptibility to HPAI H5N1 virus infections in humans could be more limited and family-based than for H7N9 (Qin et al. 2015). This could add an uncertainty in the link between human cases and the underlying circulation in poultry.

HPAI H5N1 suitability showed a much more widespread distribution than LPAI H7N9, which remains largely constrained to the southeastern coastal areas. There are many live-poultry markets in inland China (including Chengdu, Chongqing, Beijing and Shenyang cities) and these remain at relatively lower risk compared to the southeastern and coastal hotspot areas. When the new H7N9 virus emerged in China, there was an anticipation of a possible geographical expansion into other Asian countries that had many suitable areas for infection beyond China (Gilbert et al. 2014). Yet, after more than three years of seasonal and winter epidemic waves in humans, the disease has not spread much within China or internationally (or has not been observed). This may possibly relate to a yet unclear reservoir of the virus. Despite very large sampling for surveillance carried out in China, LPAI H7N9 was only rarely found in poultry farms, and most positives were from human cases traced back to live-poultry markets or from sampling carried out within live-poultry markets themselves. LPAI H7N9 virus may show some specificity toward some particular chicken breeds, such as the yellow chicken, that are only raised and traded in those areas of suitability. However, this remains very speculative, and a clear understanding of the true poultry reservoir of LPAI H7N9 is still lacking. Another likely reason of the various observed distributions of LPAI H7N9 and HPAI H5N1 may be related to the low or highly pathogenic nature of the viruses which drive differences in transmission and spread which may translate into corresponding surveillance and intervention strategies for prevention and control.

In conclusion, this study found that improvements to the poultry census data had limited impact on the outputs of suitability models for HPAI H5N1 and LPAI H7N9, that a strong and positive association between HPAI H5N1 and live-poultry markets was quantified for the first time, and that the distribution of both the HPAI H5N1 and LPAI H7N9 suitability show several areas in common, in particular in the Shanghai and Guangdong areas, which are both areas of rapid recent economic development (Yue et al. 2014).

## Electronic supplementary material

Below is the link to the electronic supplementary material.

Supplementary material 1 (DOCX 1377 kb)
