# Supplementary material for: H7N9 and H5N1 avian influenza suitability models for China: accounting for new poultry and live-poultry markets distribution data
Source: Stoch Environ Res Risk Assess. 2016 Dec 5;31(2):393–402. doi: 10.1007/s00477-016-1362-z (PMC5329093; doi:10.1007/s00477-016-1362-z)
Supplement: Supplementary file 1 — Supplementary material 1 (DOCX 1377 kb) [file 477_2016_1362_MOESM1_ESM.docx]

**H7N9 and H5N1 avian influenza suitability models for China: accounting for new poultry and live-poultry markets distribution data (Supplementary Information)**

Jean Artois, Shengjie Lai, Luzhao Feng, Hui Jiang, Hang Zhou, Xiangping Li, Madhur S Dhingra, Catherine Linard, Gaëlle Nicolas, Xiangming Xiao, Timothy P. Robinson, Hongjie Yu, Marius Gilbert

# Supplementary Information 1


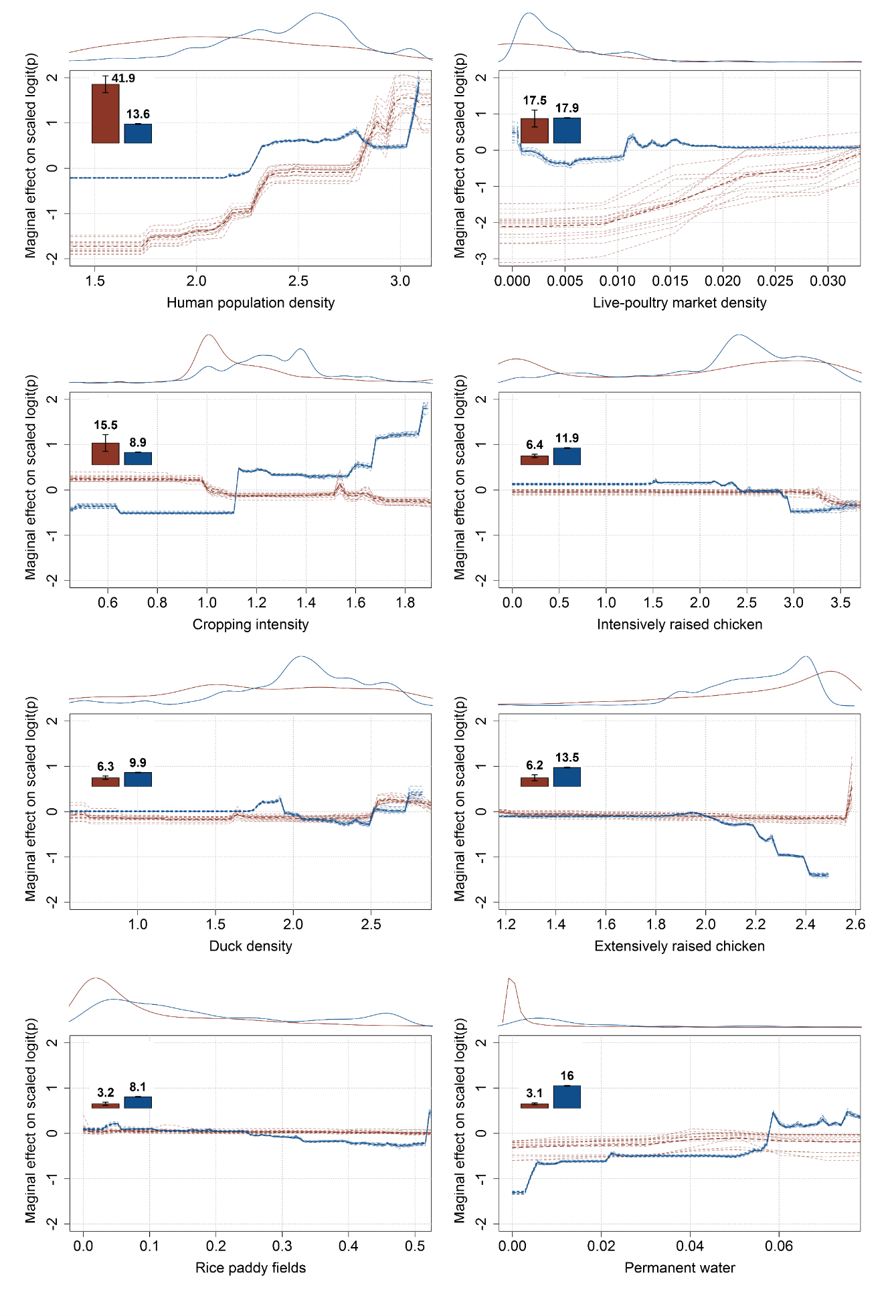


Figure A. Relative contribution (bar plots) and partial dependent plot (curves) of each predictor of the BRT models of HPAI H5N1 outbreaks (red) and LPAI H7N9 infected markets (blue). The raw number of live-poultry markets is used as predictor instead of the one modeled in Gilbert et al. (2014). The relative contribution of each predictor is scaled so that the sum of all predictor variables adds to 100%, and measures the number of times a predictor is selected for splitting the dataset over the trees. The partial dependent plot gives a graphical description of the marginal effect of a predictor on the predicted response. The opaque line represents the mean marginal effect, whilst transparent lines represent each bootstrap. On the top of each graph, the density function of the observed distribution of predictors is displayed for one bootstrap and for the two analyses (red: HPAI H5N1; blue: LPAI H7N9).


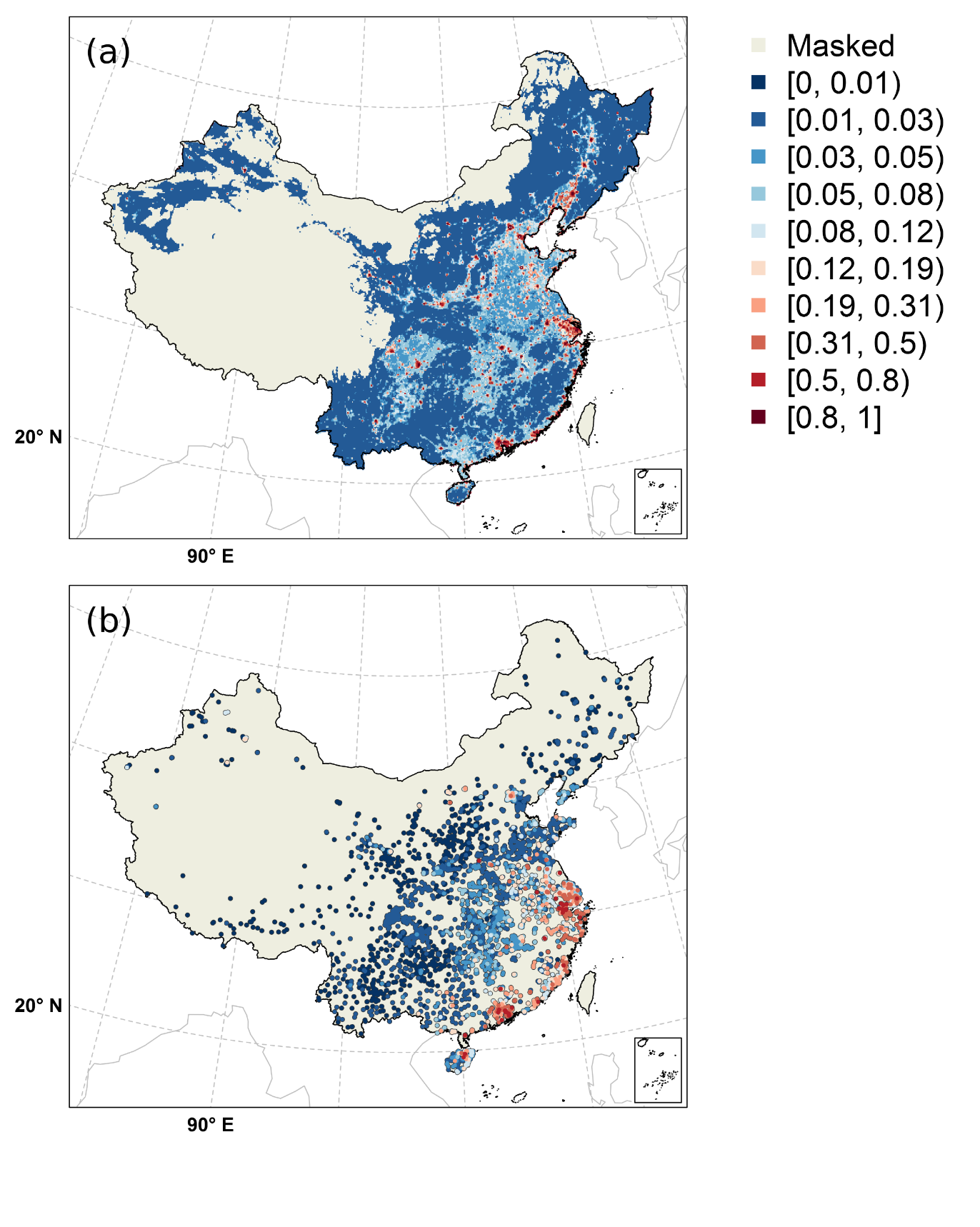


Figure B. Predicted maps of the probability of presence of HPAI H5N1 outbreaks (top) and the probability for a market of being infected by LPAI H7N9 (bottom). Note that infection risk is estimated as the probability that a pixel (HPAI H5N1) or market (LPAI H7N9) would be infected. The raw number of live-poultry markets is used as predictor instead of the one modeled in Gilbert et al. (2014). The mask corresponds to the areas where human and poultry density was lower than 5 persons, heads / km^2^. This figure was built with the R-3.3.1 software (<https://cran.r-project.org/>). The graticule is composed of a 10-degree increments and the coordinate system is ' SR-ORG:7564'.

.

# Supplementary Information 2


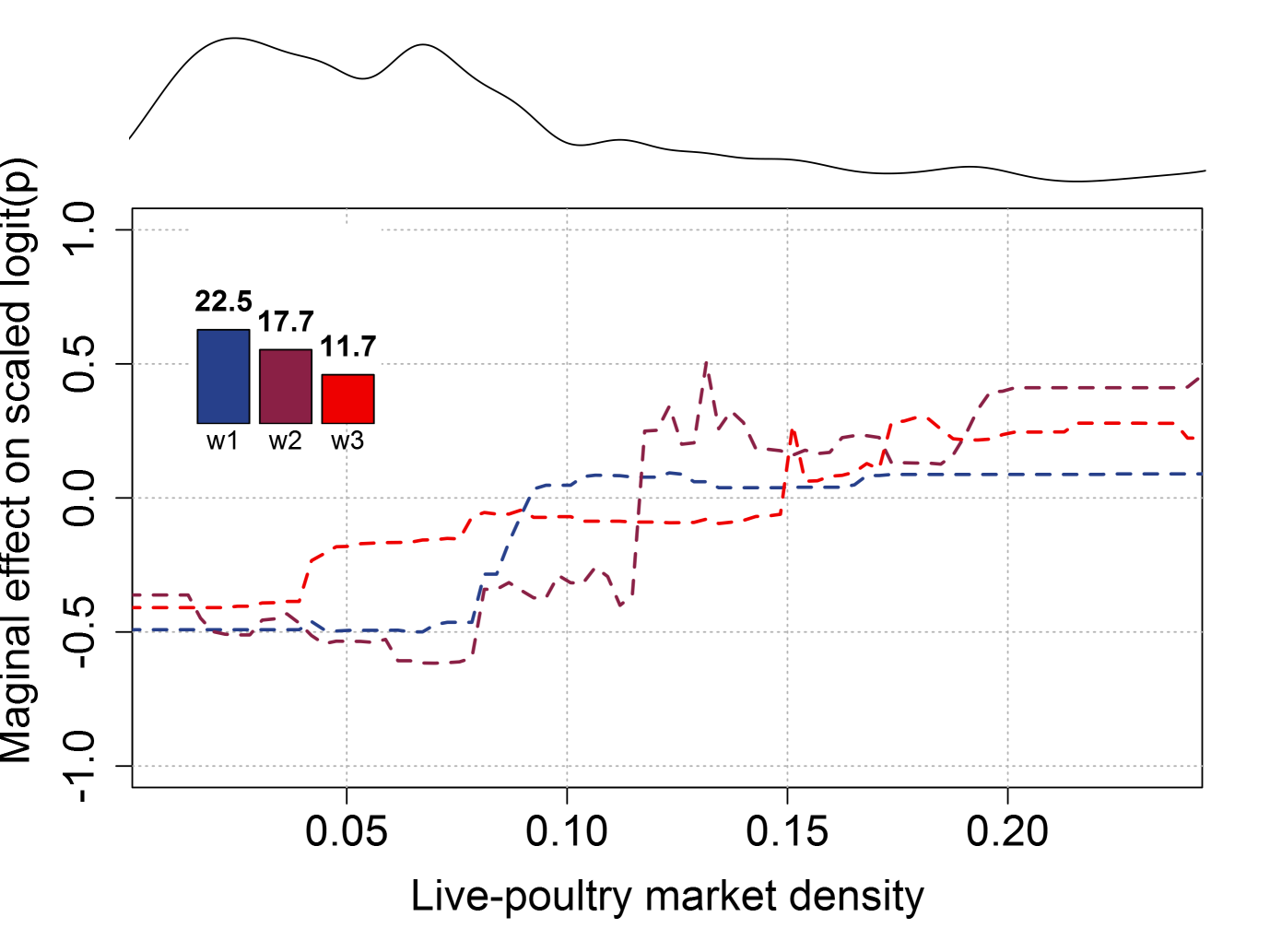


Figure A. Relative contribution (bar graph) and partial dependent plot (curve graph) of the live-poultry market density variable of the BRT models of LPAI H7N9 infected markets for the 2012/2013, 2013/2014 and 2014/2015 winters epidemic waves (abbreviated w1, w2, and w3, respectively). The relative contribution measures the number of times a predictor is selected for splitting the dataset over the trees while the partial dependent plot gives a graphical description of the marginal effect of a predictor on the response (the probability of virus presence). On the top of graph, the density function of the observed distribution of the live-poultry market variable is displayed.
